# Social immunity of the family: parental contributions to a public good modulated by brood size

**DOI:** 10.1007/s10682-015-9806-3

**Published:** 2015-11-11

**Authors:** Ana Duarte, Sheena C. Cotter, Catherine E. Reavey, Richard J. S. Ward, Ornela De Gasperin, Rebecca M. Kilner

**Affiliations:** Department of Zoology, University of Cambridge, Downing Street, Cambridge, CB2 3EJ UK; School of Biological Sciences, Queen’s University Belfast, MBC, 97 Lisburn Rd, Belfast, BT9 7BL UK; School of Life Sciences, University of Lincoln, Brayford Pool, Lincoln, Lincolnshire LN6 7TS UK

**Keywords:** Antibacterial, Lysozyme, Social evolution, Insect, Trade-off, Game theory

## Abstract

Social immunity refers to any immune defence that benefits others, besides the individual that mounts the response. Since contributions to social immunity are known to be personally costly, they are contributions to a public good. However, individuals vary in their contributions to this public good and it is unclear why. Here we investigate whether they are responding to contributions made by others with experiments on burying beetle (*Nicrophorus vespilloides*) families. In this species, females, males and larvae each contribute to social immunity through the application of antimicrobial exudates upon the carrion breeding resource. We show experimentally that mothers reduce their contributions to social immunity when raising large broods, and test two contrasting hypotheses to explain why. Either mothers are treating social immunity as a public good, investing less in social immunity when their offspring collectively contribute more, or mothers are trading off investment in social immunity with investment in parental care. Overall, our experiments yield no evidence to support the existence of a trade-off between social immunity and other parental care traits: we found no evidence of a trade-off in terms of time allocated to each activity, nor did the relationship between social immunity and brood size change with female condition. Instead, and consistent with predictions from models of public goods games, we found that higher quality mothers contributed more to social immunity. Therefore our results suggest that mothers are playing a public goods game with their offspring to determine their personal contribution to the defence of the carrion breeding resource.

**Electronic supplementary material:**

The online version of this article (doi:10.1007/s10682-015-9806-3) contains supplementary material, which is available to authorized users.

## Introduction

Social immunity, in its broadest sense, refers to any immune defence that brings benefits to others as well as the individual mounting the response itself (Cremer et al. 2007; Cotter and Kilner 2010a). Social immune systems are evident in diverse animal societies including animal families, where they are found in several fish species (Knouft et al. 2003; Giacomello et al. 2006; Little et al. 2008), frogs (Fleming et al. 2009), birds (Lafuma et al. 2001; Gwinner and Berger 2005) and insects (Kaltenpoth et al. 2005; Cardoza et al. 2006; Adams et al. 2008; Rozen et al. 2008; Cotter and Kilner 2010a). They function both to protect individuals directly and to defend a privatized resource from microbial attack (Cremer et al. 2007; Cotter and Kilner 2010a; Strassmann and Queller 2014). Furthermore, since mounting a social immune response is individually costly (e.g. Cotter et al. 2010), yet benefits the entire group (e.g. Rozen et al. 2008), social immune systems can be thought of as a type of public good (Cotter et al. 2010; Frank 2010). Recent work has revealed considerable variation in the extent to which individuals contribute to this public good, some of which can be explained by variation in individual quality and trade-offs with personal immune defence (Cotter et al. 2010; Steiger et al. 2011; Cotter et al. 2013; Joop et al. 2014). However, the social factors that regulate personal contributions to social immunity within the family remain relatively poorly understood (Cotter and Kilner 2010a; Cotter et al. 2010; Arce et al. 2013; Reavey et al. 2014). Here we investigate how offspring influence maternal contributions to the social immune defence of a key breeding resource.

We focus specifically on the social immune system of the burying beetle, *Nicrophorus vespilloides*, as our model system. Burying beetles breed upon a small vertebrate carcass, from which they shave the fur or feathers, while rolling the flesh into a ball, and burying it in a shallow grave. During this time, and also after the larvae hatch, the beetles continuously coat the carcass with antimicrobial oral and anal exudates (Rozen et al. 2008; Cotter and Kilner 2010b). Since the carcass provides both food and a nest for the beetle family, the function of antimicrobial exudates may be two-fold: to defend a crucial food resource from bacterial competitors (Rozen et al. 2008) and to promote nest hygiene, protecting larvae and parents from pathogenic bacteria (Cotter and Kilner 2010a). Current evidence indicates a relationship between the beetles’ antimicrobial exudates and disease risk. The antimicrobial potency of anal exudates from adult beetles has been found to increase in response to higher bacterial loads (Cotter et al. 2010) and to improve larval survival in the presence of pathogenic bacteria (Arce et al. 2012). Furthermore, the anal exudates of burying beetles have several features also found in immune function in insect haemolymph. So far, all evidence suggests that lysozyme, a well-known component of the insect internal immune system, is responsible for much of the anti-bacterial activity in exudates (Arce et al. 2012). Anal exudates also show phenoloxidase activity, which seemingly trades-off against lytic activity (Cotter and Kilner 2010b), a feature also shown in the immune system of several insects (e.g. Freitak et al. 2007; Cotter et al. 2008; Povey et al. 2009). The antibacterial activity of burying beetles is stimulated only when they are provided with a carcass (Cotter et al. 2010) and after reaching its peak during larval hatching, drops off during the course of the breeding event (Cotter et al. 2013), at the end of which the carcass is typically fully consumed, thus resembling very much an immune response which is mounted to overcome a microbial challenge. Furthermore, like personal immune responses, there are fitness costs associated with upregulating antimicrobial activity in the exudates (Cotter et al. 2010). For these reasons, the beetles’ anal exudates can be considered a part of a social immune response. It is this defence of a breeding resource against microbial attack that is the social immune system of interest here.

During the first 24 h of carcass preparation, the female starts laying her eggs in the surrounding soil and roughly 72 h after egg-laying, newly-hatched larvae crawl towards the carcass. As soon as they arrive, the larvae also contribute to social immune defence by producing exudates with antibacterial activity (Arce et al. 2013; Reavey et al. 2014). Larvae are fed regurgitated meat from the carcass by both parents in the hours immediately following hatching and also forage on the flesh themselves. Partial filial cannibalism (Bartlett 1987), or larval death from other sources, is common meaning that brood size at hatching typically exceeds brood size at dispersal. Roughly 5 days after hatching, larvae disperse away from the remains of the carcass to pupate in the soil and their mother departs in search of further opportunities for reproduction (Pukowski 1933; Bartlett 1988; Scott and Traniello 1990; Trumbo 1991; Scott 1998).

In our previous work, we found that when females had a larger brood at dispersal, their anal exudates exhibited lower lytic activity (or antimicrobial potency) (Cotter et al. 2013). One explanation for this finding is that it reflects a trade-off between investment in immunity and investment in fecundity (clutch size, larval provisioning, or both). Work on other species has shown that increased investment in reproduction and parental care compromises investment in personal immunity, and thus increases parasite load (Festa-Bianchet 1989; Richner et al. 1995; Deerenberg et al. 1997; Siva-Jothy et al. 1998; Gershman et al. 2010). Perhaps social immune systems are affected in a similar way.

An alternative (and novel) explanation is that mothers are playing a public goods game with their own offspring to determine their contribution to social immunity. If each individual larva produces a similar quality or quantity of antimicrobial exudates, irrespective of brood size, then collectively a large brood contributes more to the social immune defence of the carcass than does a small brood. Females raising large broods can afford to reduce their own investment in social immunity and so contribute less to the public good.

Here we experimentally investigate which of these two explanations more accurately describes maternal contributions to social immunity. This is more difficult than might at first appear because the two hypotheses yield several identical predictions (Table 1). Our approach, therefore, is to apply a combination of different experiments. For each of the two competing hypotheses, we derive a unique set of predictions (Table 1). First, we investigate whether the antibacterial activity shown by the brood is affected by brood size. A necessary condition for the public goods hypothesis to be valid is that the brood’s qualitative contribution to social immunity is either unaffected or positively affected by brood size; only then can a reduction of the maternal contribution to social immunity as a response to brood size be seen as a public goods game (Table 1). Next, we manipulate brood size (independent of clutch size), using cross-fostering experiments, to ensure that any pattern observed in maternal behaviour or social immunity is not caused by a trade-off between egg production and post-hatching investment. We focus on females because these remain longer with the larvae than males, they provide most of the direct care (Smiseth and Moore 2004), and it has been previously established that social immunity is costly in females (Cotter et al. 2010). With these experimental broods, we seek behavioural evidence of a trade-off between offspring provisioning and carcass defence by using parental time budgets to measure the effort devoted to each activity. Only the trade-off hypothesis predicts that increased time spent provisioning large broods should cause a reduction in behaviours associated with maintaining the carcass through the application of exudates. Finally, we repeat the brood size manipulation but this time we also manipulate maternal condition (by varying the extent of care that mothers received when they were larvae). We measure the antimicrobial potency of the female’s anal exudates before and after hatching. We use this second experiment to determine whether an increase in brood size causes a corresponding overall decline in maternal contribution to social immunity, as predicted by both hypotheses. The trade-off hypothesis also predicts a negative correlation between the female’s lytic activity before hatching and the mass of her brood at hatching (because investing in social immunity would reduce allocation of resources to brood mass) whereas no equivalent relationship is predicted by the public goods hypothesis. In addition, if the negative correlation between brood size and lytic activity results from a resource-based trade-off, then we predict this relationship to change with female condition because of the corresponding change in the pool of resources available within each female to sustain each activity. If, instead, maternal contributions to social immunity are governed by a public goods game, then theory predicts that mothers in better condition should contribute more to social immunity than mothers in poorer condition, all else being equal, because they are better able to bear the costs of social immunity (Frank 2010).

**Table 1:** The predicted results from each experiment for either the trade-off or public goods hypotheses

Experiments	Predictions		Results
	Trade-off hypothesis	Public goods hypothesis	
1. Relationship between brood size and brood lytic activity	No effect of brood size	No effect or positive correlation between brood size and brood lytic activity	No effect of brood size
2. Manipulation of brood size followed by behavioural observations	Increased time spent provisioning larger broods reduces time spent maintaining carcass	No trade-off	No trade-off
3. Manipulation of brood size and female condition, followed by measurement of female lytic activity	Rearing large broods causes reduction in maternal lytic activity	Rearing large broods causes reduction in maternal lytic activity	Rearing large broods causes reduction in maternal lytic activity
	Female condition affects slope/elevation of trade-off	Females in better condition show higher lytic activity	Female condition (in hours of care received as larvae) does not significantly affect lytic activity; but larger females show higher lytic activity
	Negative correlation between lytic activity of female’s exudates prior to hatching and brood mass at hatching	No such correlation	Positive correlation between lytic activity prior to hatching and brood mass at hatching

## Materials and methods

The experiments were performed throughout 2006 and in February and March 2013. We used beetles from a laboratory stock population established in 2005 at the University of Cambridge from wild beetles caught in woodlands surrounding Cambridge, and kept under standard conditions of light and temperature (see Cotter et al. 2010 for details). Every summer, field-caught beetles were added to the laboratory stock, to maintain genetic diversity. Adult beetles were kept in individual plastic boxes (12 × 8 × 2 cm) filled with moist soil and fed minced beef twice per week. When sexually mature, pairs of males and females were placed in plastic containers (17 × 12 × 6 cm) half-filled with moist soil and also containing a mouse carcass (12–16 g). Eight days later, dispersing larvae were removed, placed in plastic boxes of 5 × 5 individual divisions, with one larva per cell, covered with moist soil and left to pupate for 3 weeks.

### Experiment 1: Relationship between lytic activity of larval exudates and brood size

The methods are described in detail in Reavey et al. (2014). Briefly, sexually mature beetle pairs were provided with a mouse carcass in a standard breeding box and allowed to rear a brood. Exudates were collected from every member of the brood 3–5 days after hatching using a capillary tube, and pooled into a single sample per brood for each day of collection. 1 µl of pooled exudate per brood was used in a lytic zone assay to measure antimicrobial activity, following Cotter et al. (2010). In brief, agar was mixed with a solution of frozen cells of *Micrococcus lysodeikticus*, and plated in Petri dishes. We punched holes of approximately 1 mm diameter into the solidified agar mix and applied 1 µl of thawed exudate in each hole. We measured the diameter of the lytic zone appearing after 24 h of incubation at 33 °C, using the free software ImageJ. Egg white lysozyme at known concentrations was also applied in holes to create standard curves from which we derived the slope and intercept of the regression explaining the relationship between lytic activity (in mg/ml lysozyme equivalents) and diameter of the lytic zone.

### Experiment 2: Brood size and time budget of parental activities

We selected pairs of sexually mature virgin sisters from the stock population and paired each female with an unrelated sexually mature virgin male. We provided pairs with a piece of fresh steak (14.98 g ± 0.01 SE), instead of a thawed mouse, to control as accurately as possible for resource mass. 36 h after pairing, we removed males from the breeding boxes. 68 h after pairing, we transferred females and the prepared steaks to new containers. We searched for eggs in the original container and placed all the eggs we could find on moist filter paper where they remained until hatching. Meanwhile, at regular intervals, we examined the female’s new container for eggs. If any were present, we transferred the female and her carcass to another new container and added the additional eggs to her original set on the filter paper. When the females’ own larvae started to hatch, we selected pairs of sisters with intermediate sized clutches (29.33 eggs ± 0.40 SE), to control for egg-laying effort, and transferred them with their steak to new containers. At this point, we gave each sister either a small brood of five larvae (*N* = 20) or a large brood of 20 larvae (*N* = 20). Larvae were unrelated to the foster mother. All females accepted their foster brood.

On the day of hatching, we observed the females’ behaviour under red light to simulate underground conditions (Smiseth et al. 2005). We recorded behaviour by instantaneous scan sampling (Martin and Bateson 1986), scanning once per minute for 30 min. We scored the following behaviours: (1) Provisioning of the brood—defined as mouth to mouth contact between the parent and at least one larva. (2) Maintenance of the carcass and the crypt (the soil surrounding the carcass)—adding secretions to or manipulating the surface of the carcass, excavating the crypt or moving the carcass from below. (3) Guarding the brood—standing over or near the crater making frequent head movements from side to side. (4) Other behaviour—any behaviour other than provisioning, maintenance and guarding, this includes walking, grooming and consuming carrion. (5) Absence—being away from the crypt.

### Experiment 3: Social immunity in response to brood size and female condition

#### Manipulating female condition by changing developmental conditions

In previous work, we showed that females that received no post-hatching care as larvae subsequently raised fewer offspring upon becoming mothers themselves than females that received either 24 h or full post-hatching care (Boncoraglio and Kilner 2012, Kilner et al. 2015). Furthermore, they also suffered higher costs of reproduction (Kilner et al. 2015). We therefore chose to manipulate female condition by varying the duration of care received as larvae. We placed 20 pairs of unrelated virgin males and females from the stock population in a breeding box with a mouse carcass (23.3 g ± 2.5 SD). In half of the pairs, parents were removed 3 days after pairing, at the time of larval hatching. The developing broods thus received no post-hatching care (“0 h” broods). In the other pairs, parents were allowed to stay until day 4 after pairing (“24 h” broods). In both treatments, larvae completed their development on the carcass until dispersal and then were left to pupate under standard conditions. Upon reaching adulthood, we collected two females per family (*N* = 20 for “0 h” broods, *N* = 18 for “24 h” broods). In each family, each sister was assigned to one of two brood size treatments (“small” and “large”, see below).

#### Manipulating brood size

Three-week old “0 h” and “24 h” females were paired with virgin males from the stock population, under standard breeding conditions with a mouse carcass (12.9 g ± 2.4 SD). We removed males after carcass preparation but prior to hatching, 56 h after pairing. At larval hatching, we collected the larvae that had arrived at the carcass, weighed them, and placed the female and the carcass in a new box half-filled with fresh compost, to prevent new hatchlings arriving at the carcass after brood size manipulation. Only females whose own larvae had hatched were subsequently used (17 females from 0 h treatment, 16 females from 24 h treatment). We created foster broods from the offspring of unrelated females, who had experienced the same duration of parental care as larvae. Each female received either a small (5 larvae) or large (20 larvae) brood of unrelated larvae. All females accepted the foster brood and reared the larvae until they were ready to disperse from the carcass. At dispersal, we counted and weighed the larvae from each brood.

#### Anal exudate collection

We collected anal exudates from females at two time points: 48 h after pairing (i.e. the day before larval hatching), and 96 h after pairing [i.e. 1 day after hatching, which is when the antibacterial activity of the exudates peaks (Cotter et al. 2013)]. In this way we could account for any variation in lytic activity between females which is unrelated to the brood manipulation. Exudates are readily produced by most beetles when tapped gently on the end of the abdomen, but on rare occasions we could not collect sufficient exudates for analysis. For the subsequent statistical analysis of lytic activity results we only used females for which we had exudates at 48 and 96 h after pairing; this excluded 1 female in the ‘0 h, large brood’ treatment, 1 female in the ‘24 h, small brood’ treatment and 1 female in the ‘24 h, large brood’ treatment from the analysis. Anal exudates were kept at −20 °C until further analysis as described for experiment 1. To examine the relationship between the lytic activity of maternal anal exudates prior to hatching, and lytic activity after hatching in unmanipulated broods, we carried out a new analysis of data collected as part of a different experiment (described in Cotter et al. 2013). The beetles included in this new analysis were the controls in Cotter et al. (2013); briefly, female beetles were paired with a virgin male in a standard breeding box and provided with a mouse carcass. Males were removed after 2 days. Exudates were collected at day 2, 4 and 6 after pairing. Using the same protocol for breeding and exudate collection, females were allowed to breed repeatedly until their death. Here we analyse for each breeding event whether lytic activity at day 2 (1 day before hatching) was significantly correlated with lytic activity at day 4 (1 day after hatching).

### Statistical analyses

We used general linear (LM’s) and linear mixed models (LMM’s), with log transformations when inspection of residuals suggested heteroscedasticity or deviations from normal distribution. Starting from models including all possible covariates (such as carcass mass and female body size), we applied model selection by comparing nested models with Anova using Akaike’s Information Criterion (AIC). In all models, female family was initially included as a random effect to account for variation due to genetic or maternal effects, and only removed if it accounted for little or no variance.

When analysing average larval mass, we identified an outlier by examination of a boxplot and Cleveland dot chart of the data (Zuur et al. 2009). The outlier belonged to the ‘24 h’ and ‘small brood’ treatments, and was removed from further analysis.

For non-significant effects, we report significance upon removal from the model. For LM’s, we obtained for each fixed effect *t*-statistics and *p* values from the summary() function in R. For LMM’s, we used the “lme4” package in R (Bates et al. 2013); *t*-statistics, degrees of freedom and *p* values were calculated using Satterthwaite’s approximation, with the “lmerTest” package in R (Kuznetsova et al. 2013). Tukey post hoc comparisons were performed using the “lsmeans” package in R (Lenth 2014).

## Results

### Experiment 1: Larval lytic activity in relation to brood size

We found no significant relationship between brood size at dispersal and the brood’s collective lytic activity (Table 2; Fig.S1).

**Table 2:** LM with log-transformed larval lytic activity as response variable

	Estimate	SE	*df*	*t*	*p*
(Intercept)	0.10	0.46	74	0.21	0.83
Day	−0.55	0.12	74	−4.67	**<0.001**
Number of larvae	−0.01	0.01	74	−1.18	0.24

Significant effects (*p* value < 0.05) are shown in bold

### Experiment 2: Brood size and time budget of parental care activities

When given a large brood, females spent significantly more time guarding (LMM, slope = 0.886, SE = 0.34, *df* = 27.79, *t* = 2.615, *p* = 0.016) and feeding larvae (LMM, slope = 1.526, SE = 0.29, *df* = 19.33, *t* = 5.333, *p* < 0.001). Brood size treatment had no effect on time spent maintaining the carcass (LMM, slope = −0.120, *df* = 32, *t* = −0.474, *p* = 0.639). Furthermore, there was no overall correlation between time spent feeding and time spent maintaining the carcass (Spearman rank correlation coefficient = −0.02, *p* = 0.9, Supporting Information S2).

### Experiment 3: Social immunity in response to brood size and female condition

Antimicrobial activity increased significantly from 48 h (pre-hatching) to 96 h (post-hatching) after pairing (paired *t* test, *t* = −6.1121, *df* = 33, *p* < 0.01), as expected from previous work (Cotter et al. 2013).

Females assigned to small and large broods did not differ a priori in their pre-hatching lytic activity (Anova: *F*_1,28_ = 1.54, *p* = 0.22). Pre-hatching lytic activity was only significantly predicted by female size (LMM: slope = 1.21, SE = 0.38, *df* = 18.42, *t* = 3.18, *p* = 0.005).

**Fig. 1:**
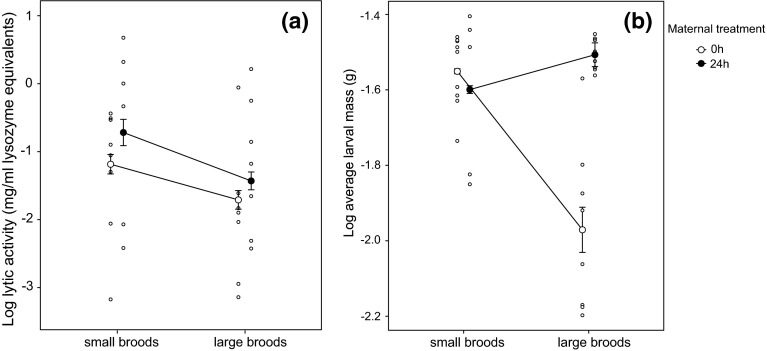
**a** Log lytic activity (in mg/ml lysozyme equivalents) of females one day after hatching. Females received either large (20 larvae, *N* = 14) or small (5 larvae, *N* = 16) broods. **b** Log average larval mass of small and large broods. In both plots *filled circles* show predicted means and standard errors of the minimal adequate model (*white circles* = 0 h females, *black circles* = 24 h females). *Open circles* are raw data. Data points from each treatment have been offset for clarity of the figure

Females that were given large broods showed on average lower levels of post-hatching antimicrobial activity than females that were given small broods (Fig. 1a; Table 3). Female size was positively correlated with post-hatching lytic activity (Table 3). Brood size treatment was only marginally significant, but its removal from the model increased AIC (from 88.7 to 90.7), hence it was retained in the minimal adequate model. Maternal condition had no effect on lytic activity (LM: slope = 0.33, SE = 0.39, *df* = 26, *t* = 0.87, *p* = 0.39), nor was there an interaction between maternal condition and brood size treatment (slope = −0.53, SE = 0.83, *df* = 12.2, *t* = −0.64, *p* = 0.53).

**Table 3:** LM with log-transformed female lytic activity 1 day after hatching as response variable, when brood size was experimentally manipulated

	Estimate	SE	*df*	*t*	*p*
(Intercept)	−7.61	2.41	27	−3.16	**0.004**
Brood (small)	0.69	0.35	27	1.98	0.058
Female size	1.22	0.48	27	2.25	**0.018**

Significant effects (*p* value < 0.05) are shown in bold

### Experiment 3: Brood performance

Brood mass at hatching, prior to brood size manipulation, did not differ between treatments (LMM, *t* = 1.24, *p* = 0.23). Contrary to the prediction of the trade-off hypothesis, we found that pre-hatching lytic activity of the biological mother significantly predicted brood mass at hatching (LMM: slope = 0.24, SE = 0.04, *df* = 29.3, *t* = 5.73, *p* < 0.001).

**Fig. 2:**
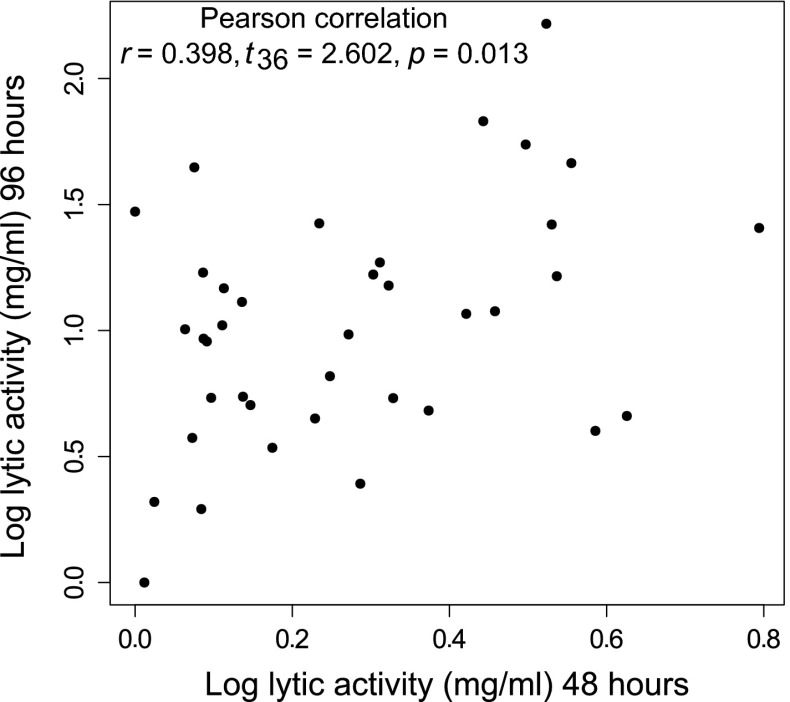
Scatterplot of log lytic activity in female exudates prior to hatching (48 h) against log lytic activity 1 day after hatching (96 h)

Furthermore, in unmanipulated broods (data corresponding to control treatment in Cotter et al. 2013) we found a significant positive correlation between pre- and post-hatching lytic activity of the mother’s anal exudates (Pearson product moment correlation: *r* = 0.398, *t*_36_ = 2.602, *p* = 0.013)(Fig. 2).

Maternal condition affected average larval mass at dispersal, with low quality mothers (i.e. those that received 0 h post-hatching care as larvae) raising larvae of lower mass than higher quality mothers (i.e. those that received 24 h post-hatching care as larvae) (LMM: slope = −0.25, SE = 0.07, *df* = 25.97, *t* = 3.36, *p* = 0.002), although there was a significant interaction between brood size and maternal condition treatments (Fig. 1b, interaction term: slope = 0.32, SE = 0.10, *df* = 17.84, *t* = 3.30, *p* = 0.004). The average mass of larvae raised by higher quality mothers did not differ significantly between large and small broods, whereas larvae raised by lower quality mothers were significantly smaller if they came from large broods rather than small broods (Tukey post hoc comparison: estimated difference = −0.39, SE = 0.06, *df* = 16.81, *t*-ratio = −6.2, *p* < 0.001). Overall, average larval mass at dispersal increased with carcass mass (LMM: slope = 0.063, SE = 0.01, *df* = 25.87, *t* = 4.28, *p* < 0.001), though the nature of this relationship differed between the brood size treatments (interaction term: slope = −0.05, SE = 0.02, *df* = 25.33, *t* = −2.59, *p* = 0.01).

## Discussion

Our aim is to explain why maternal contributions to social immunity are inversely correlated with brood size at dispersal (Cotter et al. 2013). Our first key experimental result is the finding that this relationship is caused by variation in brood size, because females given small broods of five larvae showed higher lytic activity in their anal exudates than females receiving large broods of 20 larvae. But how does this relationship arise? Does a resource-based trade-off mean that putting more investment into reproduction leaves less for social immune defence? Or are mothers adjusting their contribution to social immunity in relation to their brood’s contribution to this public good? Note that while these two hypotheses are not mutually exclusive, each yields a unique set of predictions, which allows us to determine which hypothesis is more consistent with current empirical findings.

In general, our results are not consistent with a trade-off between investment in reproduction and social immunity (see Table 1 for original predictions). We found no evidence that the negative association between brood size and maternal investment in social immunity is due to a trade-off with investment in egg production. This is because when we controlled for pre-hatching investment by mothers in their clutch, using cross-fostering in experiment 3, we still found that females raising a large brood contributed less to social immunity than females raising small broods.

Furthermore, we found that female contributions to social immunity before hatching were positively correlated with the mass of their original brood at hatching (experiment 3), as well as with social immunity post-hatching in unmanipulated broods. These positive relationships are probably driven by female quality (as found in Steiger 2013) and do not support the negative correlation predicted by the trade-off hypothesis. Hence, females do not seem to be constrained by a trade-off between pre-hatching and post-hatching investment.

We found no behavioural evidence for a trade-off either. Females spent approximately the same amount of time maintaining the carcass, regardless of their brood size, even though they spent more time feeding larger broods.

In another experimental approach, we tried to expose any trade-off between investment in reproduction and social immunity by manipulating female condition, so changing the hypothetical pool of resources to be divided between each action. The manipulation worked, in the sense that females in poorer condition were less capable of investing in large numbers of offspring, producing smaller larvae when rearing large broods (Fig. 2b). If social immunity and brood size were trading-off, we would expect females in poor condition to show this trade-off more markedly than females in better condition (van Noordwijk and de Jong 1986). Yet, we found that female condition did not affect the relationship between social immunity and brood size (Fig. 2a). Instead, females in good and poor condition adjusted their lytic activity in a similar way in response to our manipulations of brood size.

Yet further evidence against the trade-off hypothesis comes from previous work where we induced females to up-regulate the lytic activity of their anal exudates by exposing carcasses to a bacterial challenge (Cotter et al. 2010). If there were a simple trade-off between investment in social immunity and brood size then we would expect to observe smaller brood sizes when females were forced to invest more in social immunity—but no such change was observed (Cotter et al. 2010). Likewise, when we previously forced females to down-regulate their investment in social immunity by experimentally up-regulating their investment in personal immunity (Cotter et al. 2013), we found that down-regulation of social immunity alone was not coupled to an increase in brood size, again demonstrating little support for a trade-off between investment in brood provisioning and social immunity.

Thus, the best explanation for the negative relationship between brood size and maternal contributions to social immunity we currently have is that females and their broods are using social cues to adjust their respective contributions to a public good. The two lines of evidence that support this interpretation are more circumstantial than direct. First, we carried out new analyses of some of the data published in Reavey et al. (2014), to investigate the association between brood size and the brood’s contribution to social immunity. We predicted that the lytic activity of larval exudates should either stay constant or rise with increasing brood size, since the lytic activity of maternal exudates falls with increasing brood size. Finding a negative correlation between larval lytic activity and brood size would refute the public goods hypothesis. We found the lytic activity of the brood to be constant regardless of brood size.

The second line of indirect support for the suggestion that maternal contributions to social immunity are governed by a public goods game comes from our test of the prediction from public goods theory that better quality mothers should produce exudates with greater lytic activity (Frank 2010). Although we did not find that our experimental manipulation of maternal condition influenced females’ contributions to social immunity, we did find that larger females produced exudates that were more potently antimicrobial after their larvae hatched. Whether this is because larger females were better able to sustain the higher fitness costs associated with producing exudates with greater lytic activity, as assumed theoretically (Frank 2010), remains to be determined in future work.

While the current work does not offer the definitive answer on whether beetle mothers and offspring are involved in a public goods game over their contributions to social immunity, it does suggest that the simplest explanation (a trade-off between social immunity and brood size) is not the most likely. As we learn more about the genes underlying social behaviour (e.g. Cunningham et al. 2015), the definitive answer could be provided by experiments making use of the recent technological advances in genome editing, such as CRISPR/Cas (Gaj et al. 2013). Genetically engineering larvae lacking antimicrobial activity would allow us to determine to what extent maternal contributions to social immunity depend on offspring contributions.

## Electronic supplementary material

Supplementary material 1 (DOCX 52 kb)

Supplementary material 2 (DOCX 42 kb)
