# Supplementary material for: Social immunity of the family: parental contributions to a public good modulated by brood size
Source: Evol Ecol. 2015 Nov 11;30:123–35. doi: 10.1007/s10682-015-9806-3 (PMC4750363; doi:10.1007/s10682-015-9806-3)

**Figure S1.** No relationship between log antibacterial activity (in mg/ml lysozyme equivalents) of 1 µl of exudates pooled from all of the larvae in a single brood against the number of larvae in that brood at dispersal. If the antibacterial activity in individual larvae decreased with brood size a negative relationship would be expected.

**
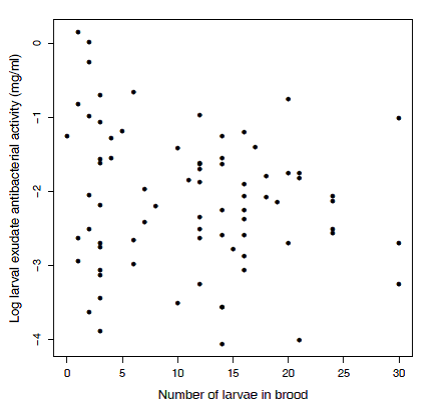
**

**Figure S2**. No trend in variance in log antibacterial activity across the range of observed brood sizes.


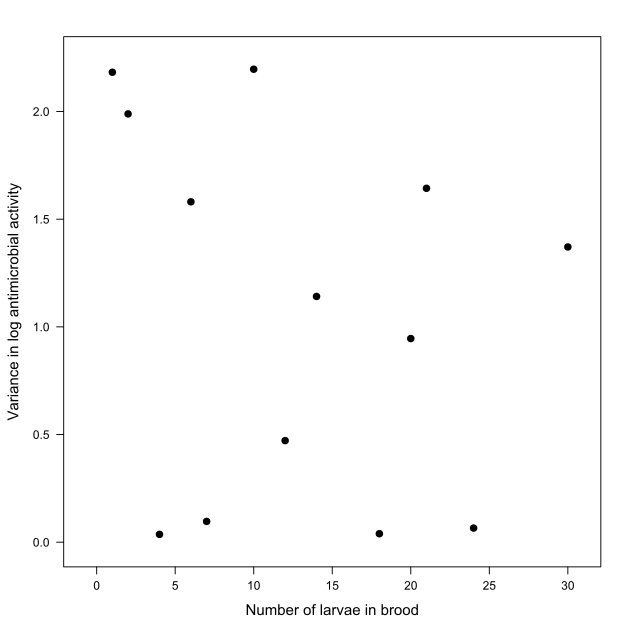

Supplement: Supplementary file 1 — Supplementary material 1 (DOCX 52 kb) [file 10682_2015_9806_MOESM1_ESM.docx]
