# Supplementary material for: Social immunity of the family: parental contributions to a public good modulated by brood size
Source: Evol Ecol. 2015 Nov 11;30:123–35. doi: 10.1007/s10682-015-9806-3 (PMC4750363; doi:10.1007/s10682-015-9806-3)

**Figure S3.** No relationship between the proportion of time spent provisioning and proportion of time spent maintaining carcass (Spearman rank correlation coefficient = -0.02, *p* = 0.9). Number of overlapping data points is indicated by size of circles.


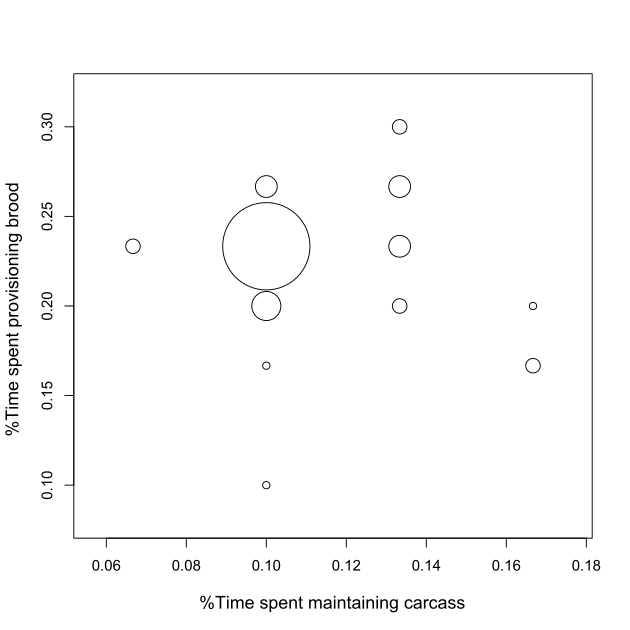

Supplement: Supplementary file 2 — Supplementary material 2 (DOCX 42 kb) [file 10682_2015_9806_MOESM2_ESM.docx]
